# A curated global checklist of polychaetes (Annelida) from deep‑sea chemosynthetic and reducing habitats

**DOI:** 10.3897/BDJ.14.e212692

**Published:** 2026-10-27

**Authors:** Yu Li, Yun Chen, Xuwen Wu

**Affiliations:** 1 Laboratory of Marine Organism Taxonomy and Phylogeny, Shandong Province Key Laboratory of Marine Biodiversity and Bio-resource Sustainable Utilization, Institute of Oceanology, Chinese Academy of Sciences, Qingdao, China Laboratory of Marine Organism Taxonomy and Phylogeny, Shandong Province Key Laboratory of Marine Biodiversity and Bio-resource Sustainable Utilization, Institute of Oceanology, Chinese Academy of Sciences Qingdao China; 2 University of Chinese Academy of Sciences, Beijing, China University of Chinese Academy of Sciences Beijing China; 3 Meilianzhonghe Veterinary Hospital, Qingdao, China Meilianzhonghe Veterinary Hospital Qingdao China

**Keywords:** Annelida, checklist, chemosynthetic habitat, cold seep, dataset, deep sea, hydrothermal vent, polychaetes, reducing habitat, whale fall, wood fall, WoRMS

## Abstract

**Background:**

Deep‑sea chemosynthetic and reducing habitats, including hydrothermal vents, cold seeps, methane seeps, whale falls, wood falls, organic falls and other reducing sedimentary environments, represent key systems for deep‑sea biodiversity research. Polychaetes are a species‑rich and taxonomically well‑studied benthic group in these environments. However, relevant records are scattered across taxonomic papers, regional surveys, ecological studies, monographs, reviews and databases. No dedicated global dataset is currently available with unified taxonomic validation, habitat coding and literature provenance.

**New information:**

This paper introduces a publicly released curated global checklist dataset of polychaetes from deep‑sea chemosynthetic and reducing habitats (version 1.0.1). Compiled from published literature, the dataset harmonises scientific names, accepted names under the World Register of Marine Species (WoRMS), AphiaIDs, LSIDs and taxonomic status. The dataset is archived on Zenodo (https://doi.org/10.5281/zenodo.22011206) and contains 368 checklist‑level taxonomic records, amongst which 363 records are included in core statistics according to the 'includeInCoreStatistics' field. It provides taxonomic hierarchies, WoRMS identifiers, standardised and raw habitat descriptions, locality information, source references, plus taxonomic and ecological notes. Supplementary materials include a reference table, habitat controlled vocabulary, field dictionary, README, CITATION file, licence and version history. The dataset supports taxonomic syntheses, global‑ or regional‑scale biodiversity overviews, broad‑scale habitat‑association analyses and subsequent compilation of occurrence‑level data. It is not suitable for fine‑scale species distribution modelling, station‑level community analyses or sampling‑effort‑corrected richness estimation.

## Introduction

Hydrothermal vents, cold seeps and methane seeps sustain chemosynthesis via sulphide, methane, hydrogen and other reduced compounds (Weiss et al. 1977, Corliss et al. 1979, Paull et al. 1984). Whale falls, wood falls and other large organic falls develop reducing microenvironments during decomposition and can support chemosynthesis‑associated communities at certain successional stages (Smith et al. 1989, Bienhold et al. 2013). Despite differences in origin, persistence and geochemistry, these habitats collectively form a distinct system of reducing deep‑sea environments (Levin et al. 2007).

Polychaetes constitute one of the most important benthic faunal components in deep‑sea chemosynthetic and reducing habitats, encompassing multiple lineages, such as Polynoidae, Dorvilleidae and Siboglinidae (Rouse et al. 2004, Desbruyères et al. 2006, Hilário et al. 2011). Records accumulated over decades are dispersed amongst species descriptions, regional surveys, ecological investigations, handbooks and reviews. Publications adopt inconsistent taxonomic frameworks, genus‑species combinations, habitat terminology and record granularity. Some historical names have undergone synonymisation or generic re-assignment and certain habitat associations have been recognised only in later studies. Such discrepancies hamper direct comparison and reuse of global datasets.

Major global biodiversity resources relevant to marine data include WoRMS, OBIS and GBIF. WoRMS provides standardised taxonomic information, but does not offer a dedicated, consistently curated habitat framework for polychaetes from deep-sea chemosynthetic and reducing environments. OBIS and GBIF primarily host occurrence‑level data with highly variable habitat descriptors (GBIF.org 2026, OBIS 2026, WoRMS Editorial Board 2026). Authoritative traditional checklists are often restricted to specific habitats or time snapshots and are not readily updatable. None of these resources currently fulfils the role of a dedicated, line‑by‑line curated checklist for polychaetes of deep‑sea chemosynthetic and reducing habitats with unified habitat coding and literature linking. This paper describes a curated global checklist dataset (version 1.0.1) for polychaetes from these environments, detailing its construction workflow, data structure, quality control, coverage and intended applications. Built upon WoRMS taxonomy, the dataset adds manually curated unified habitat codes, source references and uncertainty annotations, bridging taxonomic databases, occurrence repositories and traditional taxonomic compilations. This version prioritises checklist‑level harmonisation of scientific names, habitat associations and literature provenance, providing a foundation for future occurrence‑level curation and global syntheses.

## General description

### Purpose

This dataset provides a globally curated, WoRMS-standardised checklist of polychaetes from deep-sea chemosynthetic and reducing habitats, suitable for taxonomic synthesis, regional diversity overviews and comparative habitat-association analyses.

### Additional information

The formal title of the dataset is A Global Checklist of Polychaetes (Annelida) from Deep-Sea Chemosynthetic and Reducing Habitats, authors Yu Li and Xuwen Wu, version 1.0.1, deposited in Zenodo (doi: 10.5281/zenodo.22011206), under Creative Commons Attribution 4.0 International Licence (CC BY 4.0).

The dataset is a checklist-level data product; each row represents a taxon, not an individual station, sampling event or occurrence record. The main file is 'taxon_checklist.csv'; supplementary files include 'references.csv', 'habitat_vocabulary.csv', 'field_dictionary.csv', 'taxonomic_notes.csv', 'README.md', 'CITATION.cff', 'LICENSE.txt' and 'CHANGELOG.md'. An Excel workbook provides a human-readable version corresponding to the CSV data.

The dataset adopts a modular structure that separates taxonomic units, source references, habitat vocabulary, field definitions and special-record notes. This design facilitates both manual verification and machine reading by R, Python, databases and biodiversity data platforms, while maintaining a stable data structure for future version updates.

## Sampling methods

### Study extent

Records included in this dataset must belong to Polychaeta (Annelida) and have verifiable literature support indicating that they originate from, or are stably associated with, deep-sea hydrothermal vents, cold seeps, methane seeps, whale falls, wood falls, organic falls, experimental organic substrates or other reducing environments. Records of taxonomic or historical value whose status is not fully resolved have been retained and are flagged via 'recordType', 'taxonStatus', 'habitatCertainty' and the notes files. Records with qualifiers such as cf. are retained as provisional identifications only when they can be reasonably matched to an accepted taxon in WoRMS.

Non-polychaete records, records that cannot be confirmed as associated with the target habitats, records with untraceable sources, duplicate records adding no new information, records known only from ordinary shallow-water or ordinary deep-sea non-reducing environments and ambiguous records that cannot be reasonably linked to an available name system are excluded from the main table.

### Sampling description

The dataset uses only published materials. Literature searches were primarily conducted in Web of Science and supplemented by Google Scholar, the Biodiversity Heritage Library, publisher websites and the reference lists of key publications. Sources include original taxonomic descriptions, taxonomic revisions, hydrothermal vent and cold seep faunal studies, whale-fall and wood-fall community studies, regional surveys, ecological studies, monographs, handbooks and reviews. An iterative combination of searching, taxonomic tracking and manual verification was applied; search terms covered Polychaeta, Annelida, hydrothermal vent, cold seep, methane seep, whale fall, wood fall, sunken wood, organic fall, reducing sediment, chemosynthetic habitat and reducing habitat, supplemented by backward citation tracking from key references. The final literature search and dataset update were completed on 19 August 2026. All records were initially compiled by Yu Li and subsequently reviewed by Xuwen Wu for taxonomy and habitat assignment; disagreements were resolved by re-examining the original literature and reaching consensus. For records with complex taxonomic histories or unclear habitat assignments, efforts were made to trace back to original sources and uncertainty annotations were retained.

All scientific names were checked against WoRMS, recording 'scientificName', 'scientificNameAccepted', 'authorityAccepted', 'AphiaID', 'scientificNameID' and 'taxonStatus'. 'scientificName' represents the primary name used in the dataset, while 'scientificNameAccepted' represents the current WoRMS accepted name and 'scientificNameID' is the LSID in WoRMS. For cf. records, the original spelling is retained in 'verbatimScientificName' and the qualifier is stored in 'identificationQualifier'. WoRMS is used for name and status harmonisation, but does not replace the judgement of original literature and habitat context.

The dataset retains two taxon inquirendum, one alternative representation and one unaccepted record for taxonomic tracking, but these are excluded from core statistics. Among the four cf. provisional identifications, three are included as provisional core records in core statistics, while one is excluded because it simultaneously involves an alternative representation. An additional 12 records are flagged as suspected additional taxa, indicating that the literature suggests possible undescribed or cryptic taxa, but they are not counted as additional species. Among the three records with 'habitatCertainty = uncertain', *Flascarpia alvinae* and *Lamellibrachia victori* are retained as uncertain habitat records; *Capitella capitata* is excluded from core statistics due to instability in both taxonomic concept and habitat evidence. All above special-status entries are further explained via 'recordType', 'includeInCoreStatistics' and the 'taxonomic_notes.csv' file.

The dataset uses a controlled vocabulary to standardise habitat names, including 'hydrothermal vent', 'cold seep', 'methane seep', 'whale fall', 'wood fall', 'cow fall', 'organic fall', 'experimental substrate', 'reducing sediment' and 'uncertain'. For summary visualisation, the ten standardised habitats are further grouped into six broad categories: Hydrothermal Vent Habitat, Cold Seep Habitat, Organic Fall Habitat, Reducing Habitat, Experimental/Artificial Habitat and Uncertain Habitat. Cold Seep Habitat includes 'cold seep' and 'methane seep'; Organic Fall Habitat includes 'whale fall', 'wood fall', 'cow fall' and 'organic fall'. Original habitat descriptions are retained; standardised values are used for statistics and comparisons and 'habitatCertainty' indicates the reliability of evidence. A single taxon may be associated with multiple habitats, with multiple values separated by semicolons; this structure represents a checklist-level habitat association and does not equate to multiple occurrence records.

Each main-table record is linked via 'referenceIDs' to source references in 'references.csv'. The reference table records 'referenceID', full citation, year and DOI (if available); multiple references for one taxonomic record are indicated by semicolon-separated IDs. Judgements on taxonomic treatment, habitat uncertainty and special records are centrally documented in 'taxonomic_notes.csv', thus creating verifiable record-level linkages amongst names, habitats and sources.

### Quality control

Quality control was applied throughout the dataset construction process, including: confirming that the main table contains only polychaete records; verifying consistency of WoRMS names, accepted names, AphiaIDs, LSIDs and taxonomic status; checking uniqueness of 'taxonRecordID' and major taxonomic identifiers; validating complete correspondence of 'referenceIDs' to 'references.csv'; confirming that 'habitatStandardized' uses only controlled vocabulary values; and conducting separate manual reviews of cf., taxon inquirendum, alternative representation, unaccepted, suspected additional taxa and uncertain habitat records. CSV files use UTF‑8 encoding; the Excel file is provided for human browsing; field meanings, null-value rules and special treatments are clearly stated in the accompanying documentation.

## Geographic coverage

### Description

**Figure 1.**
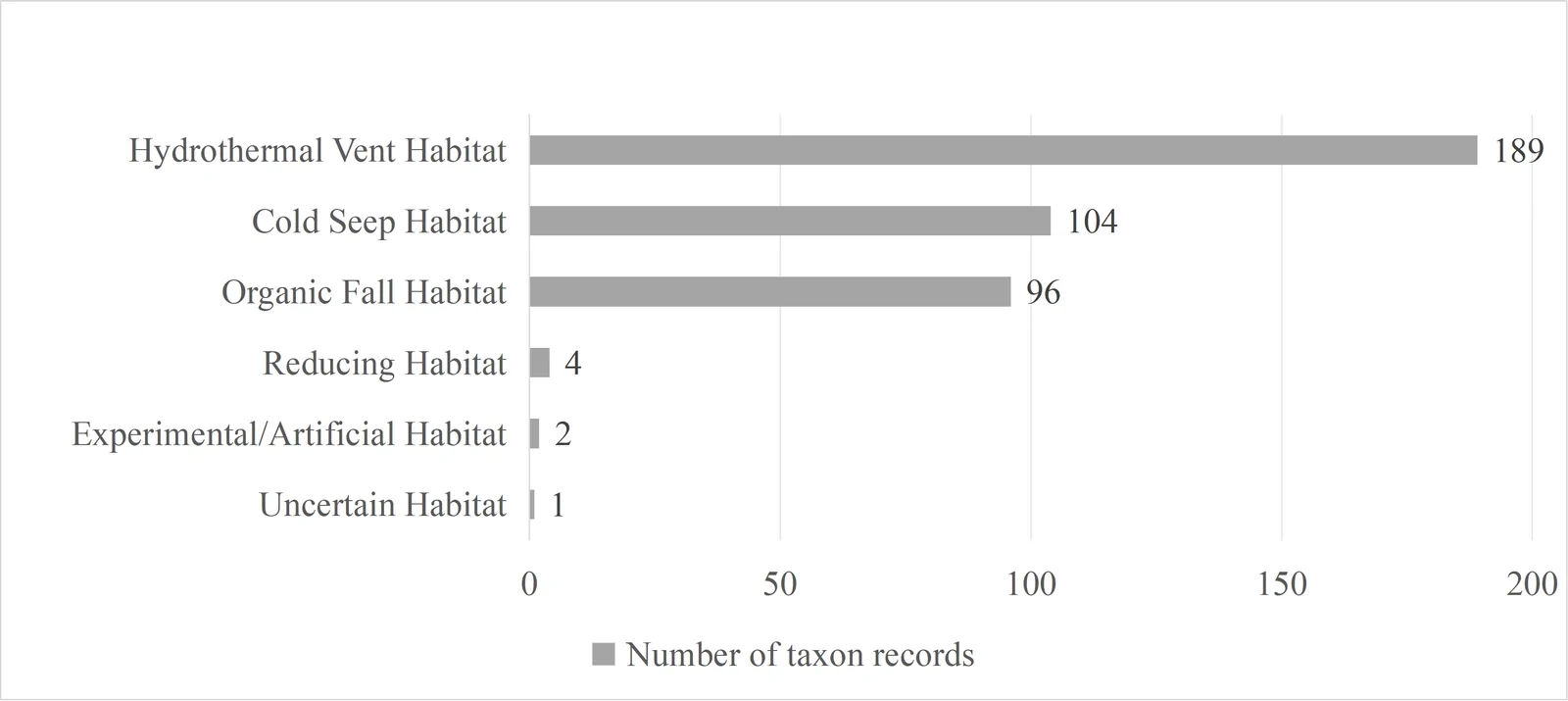
Number of taxon records associated with each habitat group.

Locality records in the dataset cover multiple major hydrothermal vent fields, cold seep areas, whale-fall and wood-fall habitats worldwide. Amongst all 368 records, the numbers of taxon records associated with individual habitat types are as follows: hydrothermal vent 189, cold seep 89, whale fall 69, wood fall 21, methane seep 15, cow fall seven, organic fall four, reducing sediment four, experimental substrate two and uncertain one. For a more intuitive display of the overall diversity pattern, Fig. 1 groups the above fine-scale habitats into six broad categories for visualisation; a taxon is counted only once within the same broad category even if it occurs in multiple fine-scale habitats under that category (Fig. 1). Since one taxon may be associated with multiple habitats, the sum across categories exceeds the total number of main-table records. These numbers represent taxon–habitat associations, not occurrence frequencies, individual counts or true abundances.

Geographic information covers major deep-sea chemosynthetic and reducing habitats worldwide, including the Pacific, Atlantic, Indian Oceans, the Southern Ocean and associated marginal seas. The location field may contain specific habitat areas, basins, ridges, trenches, marginal seas or broader regions. For records with precise station information, original geographic descriptions are retained; for historical records providing only regional names, the original wording is kept without speculative geolocation.

Therefore, this dataset is suitable for global-scale taxonomic and biogeographic data integration, but it is not an occurrence-level geographic database and is not recommended for species distribution modelling or sampling-density analyses.

## Taxonomic coverage

### Description

The main table contains 368 polychaete taxonomic records, of which 364 are WoRMS accepted records, two are taxon inquirendum, one is an alternative representation and one is an unaccepted record. According to 'includeInCoreStatistics', 363 records are included in core statistics, comprising 360 core records and three provisional core records; the remaining five records are excluded due to taxonomic or habitat evidence issues. The dataset covers multiple major polychaete families in deep-sea chemosynthetic and reducing habitats; full taxonomic hierarchy comes from WoRMS matching results. Where intermediate ranks are empty, that rank is unavailable, not applicable or not used in the adopted classification system.

**Figure 2.**
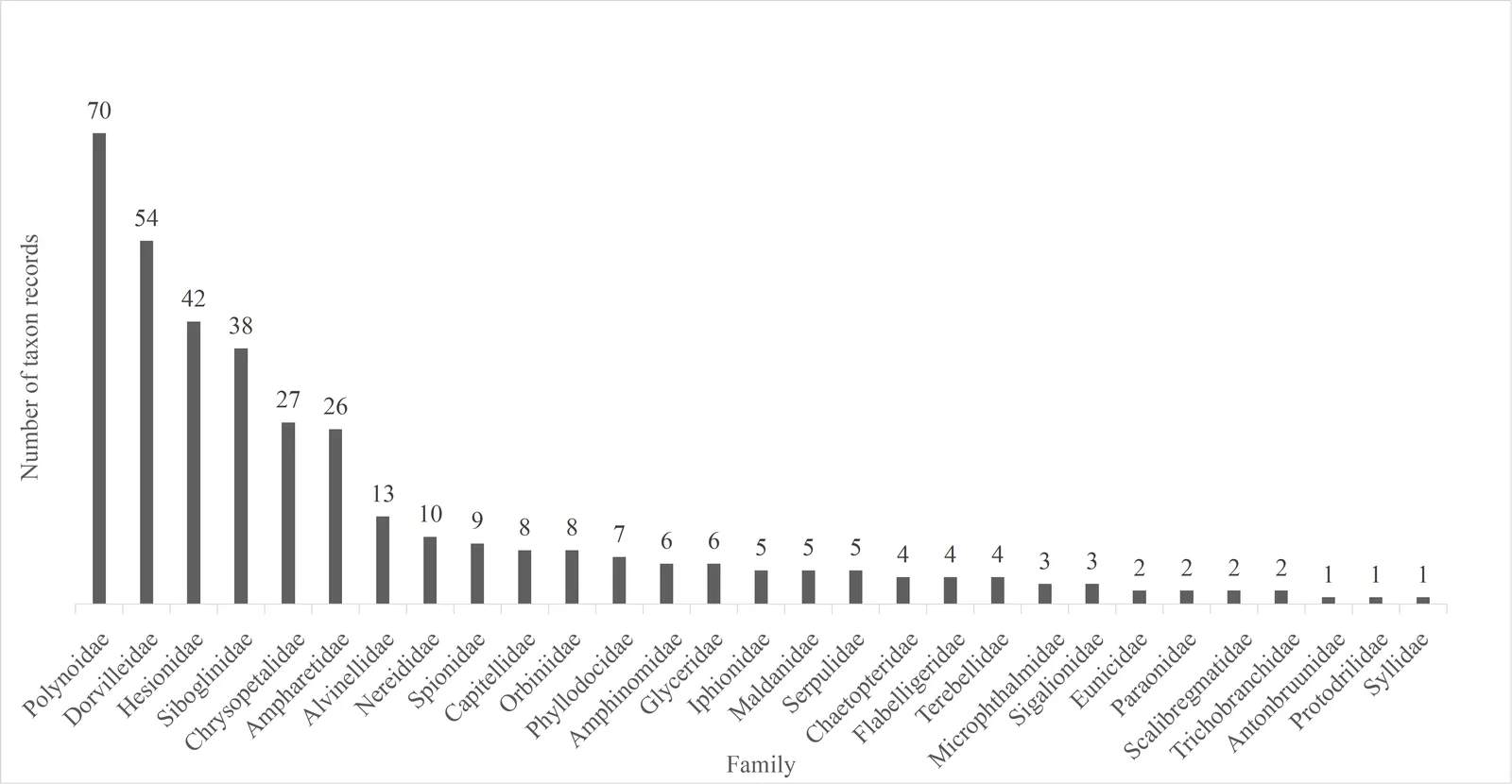
Family-level composition of the 368 polychaete taxon records.

The dataset covers the major polychaete lineages reported from deep-sea chemosynthetic and reducing habitats to date. Based on all 368 records, it involves six orders, 29 families and 123 genera, including well-represented families such as Polynoidae, Siboglinidae, Dorvilleidae and Alvinellidae, as well as a range of other polychaete families reported from reducing habitats. Fig. 2 shows the relative family-level counts in the dataset, including accepted records, provisional records, and taxonomically problematic records (Fig. 2).

## Usage licence

### Usage licence

Other

### IP rights notes

Creative Commons Attribution 4.0 International (CC-BY-4.0)

## Data resources

### Data package title

A Global Checklist of Polychaetes (Annelida) from Deep‑Sea Chemosynthetic and Reducing Habitats

### Resource link

https://zenodo.org/records/22011206

### Number of data sets

1

### Data set 1.

#### Data set name

A Global Checklist of Polychaetes (Annelida) from Deep-Sea Chemosynthetic and Reducing Habitats

#### Data format

CSV

#### Character set

UTF‑8

#### Description

The data package has been deposited in Zenodo (version 1.0.1; https://doi.org/10.5281/zenodo.22011206). The dataset is organised in a modular manner, consisting of multiple interrelated data files (Table 1).

## Additional information

### Literature coverage

This dataset was compiled, based on 161 unique published references spanning the period from 1957 to 2026. These references encompass a wide range of literature types, including original taxonomic descriptions, taxonomic revisions, regional faunal surveys and checklists, amongst others. Each reference is linked to the corresponding taxonomic records in the main table through the 'referenceIDs' field. The complete reference list is provided in the accompanying file 'references.csv'.

Before the 1980s, taxonomic research on polychaetes associated with deep-sea chemosynthetic and reducing habitats was still limited in scope. The dataset contains eight references from this period, primarily addressing traditional morphological taxonomy, regional faunal surveys and taxonomic revisions (Hartman 1957, Pettibone 1957, Southward 1961, Webb 1965, Southward 1972, Land and Nørrevang 1975, Hartmann-Schröder 1977, Oug 1978). Hartman (1957) reviewed four families — Orbiniidae, Apistobranchidae, Paraonidae and Longosomidae — and described new taxa while documenting their taxonomy and distribution. Pettibone (1957) revised the genera of Orbiniidae from North America. Southward (1961), Webb (1965), Southward (1972) and Land and Nørrevang (1975) described siboglinid tubeworms, members of which were later recognised as prominent components of deep-sea chemosynthetic and reducing habitats.

Following the discovery of hydrothermal vents in 1977, research on polychaetes inhabiting deep-sea chemosynthetic and reducing habitats gradually expanded. The dataset includes 28 references published between 1980 and 1999 (Davis and Spies 1980, Fauchald and Hancock 1981, Zottoli 1982, Blake 1985, Mañé-Garzón and Montero 1985, Pettibone 1985, Desbruyères and Laubier 1986, Desbruyères and Laubier 1988, Kuznetsov and Levenstein 1988, Desbruyères and Laubier 1989, Pettibone 1989a, Pettibone 1989b, Blake 1990, Pettibone 1990, Blake 1991, Desbruyères and Laubier 1991, Miura and Hashimoto 1991, Southward 1991, Ivanov and Selivanova 1992, Kiseleva 1992, Blake 1993, Kupriyanova 1993, Pettibone 1993, Miura and Hashimoto 1996, Hilbig 1997, Miura 1997, Desbruyères and Toulmond 1998, Nishi et al. 1999). Pettibone (1985) described new genera and species of Polynoidae from the Galápagos hydrothermal vents and the 21°N hydrothermal vent field. Blake (1985) provided a systematic account of polychaetes from hydrothermal vents in the eastern Pacific, covering multiple families, including Dorvilleidae, Hesionidae, Nereididae, Glyceridae, Orbiniidae and Maldanidae. Desbruyères and Laubier (1986) established the family Alvinellidae. Zottoli (1982) documented Ampharetidae in organic-enrichment and organic-fall environments. Davis and Spies (1980) investigated benthic communities associated with natural petroleum seeps off California, while Pettibone (1989a) recorded polychaetes from cold seeps in the Gulf of Mexico. After 1990, the number of references associated with cold seeps and whale falls increased. Miura and Hashimoto (1991) described a polychaete belonging to Maldanidae from cold seeps in Sagami Bay, Japan, whereas Miura and Hashimoto (1996) documented polychaetes belonging to Nautiliniellidae (now treated as a junior synonym of Chrysopetalidae) from cold seeps and hydrothermal vents in the Okinawa Trough and other sites around Japan. Desbruyères and Toulmond (1998) described Hesiocaeca methanicola from methane hydrates in the Gulf of Mexico, an important record of Hesionidae from a cold-seep habitat. Blake (1993) described new genera of Nautiliniellidae from cold seeps along the Florida Escarpment. Geographically, the literature covered the eastern Pacific, the western Pacific, including Japan and the Okinawa Trough, the Atlantic, including the Gulf of Mexico and the margins of the Indian Ocean, including the Java Trench. In terms of taxonomic composition, the taxa involved include Polynoidae, Alvinellidae, Siboglinidae, Maldanidae, Hesionidae and Chrysopetalidae.

Most of the references in the dataset were published between 2000 and 2026 (Blake 2000, Smirnov 2000, Gardiner et al. 2001, Watson 2001, Kojima et al. 2002, Gardiner and Hourdez 2003, Hourdez and Daniel 2003, McMullin et al. 2003, Sigvaldadóttir and Desbruyères 2003, Andersen et al. 2004, Dahlgren et al. 2004, Dreyer et al. 2004, Glover et al. 2005, Desbruyères et al. 2006, Fujikura et al. 2006, Kojima et al. 2006, Miura and Kojima 2006, Salazar-Vallejo and Orensanz 2006, Nishi and Rouse 2007, Ravara et al. 2007, Bastida-Zavala 2008, Nishi 2008, Pleijel et al. 2008, Blake 2009, Böggemann 2009, Fiege and Bock 2009, Paxton and Morineaux 2009, Quiroga and Sellanes 2009, Reuscher et al. 2009, Wiklund et al. 2009a, Wiklund et al. 2009b, Bright and Lallier 2010, Kupriyanova et al. 2010, Quiroga and Levin 2010, Watanabe et al. 2010, Aguado and Rouse 2011, Kongsrud and Rapp 2011, Reuscher et al. 2011, Southward et al. 2011, Pleijel et al. 2012, Thiel et al. 2012, Wiklund et al. 2012, Borda et al. 2013, Eichinger et al. 2013, Imajima et al. 2013, Stiller et al. 2013, Taboada et al. 2013, Artüz et al. 2014, Rubin-Blum et al. 2014, Salvo et al. 2014, Taboada et al. 2014, Georgieva et al. 2015, Plouviez et al. 2015, Ravara et al. 2015, Summers et al. 2015, Taboada et al. 2015, Reuscher and Fiege 2016, Silva et al. 2016, Watson et al. 2016, Eilertsen et al. 2017, Gaudron et al. 2017, Goffredi et al. 2017, Karaseva et al. 2017, Kongsrud et al. 2017, Queirós et al. 2017, Ravara et al. 2017, Shimabukuro et al. 2017, Zhang et al. 2017, Zhou et al. 2017, Eilertsen et al. 2018, Kobayashi and Araya 2018, McCowin and Rouse 2018a, McCowin and Rouse 2018b, Rouse et al. 2018a, Rouse et al. 2018b, Sun et al. 2018, Wu and Xu 2018, Zhang et al. 2018, Zhou et al. 2018, Jimi et al. 2019, Lindgren et al. 2019, McCowin et al. 2019, Ravara et al. 2019, Saeedi et al. 2019, Shimabukuro et al. 2019, Wu et al. 2019, Zhou et al. 2019, Blake 2020, Blake and Ramey-Balcı 2020, Hatch et al. 2020, Salazar-Vallejo 2020, Shimabukuro et al. 2020, Wang et al. 2020, Yen and Rouse 2020, Han et al. 2021, Ravara et al. 2021, Rouse and Kupriyanova 2021, Alalykina and Polyakova 2022, Jimi et al. 2022, Kim et al. 2022, Magalhães and Hilliard 2022, Pearson and Rouse 2022, Xu et al. 2022, Bergamo et al. 2023, Georgieva et al. 2023a, Georgieva et al. 2023b, Han et al. 2023, Kamei et al. 2023, McCowin et al. 2023, Sui et al. 2023, Wu et al. 2023, Zhang et al. 2023, Zhou et al. 2023, Chen et al. 2024, Hui et al. 2024, Meca et al. 2024, Villalobos-Guerrero et al. 2024, Wang et al. 2024, Watson et al. 2024, Blake 2025, Hiley et al. 2025, Kroesche and Rouse 2025, Seid et al. 2025, Sun et al. 2025, Carrerette et al. 2026). The application of molecular phylogenetic methods became increasingly common during this period. For example, Borda et al. (2013), Lindgren et al. (2019) and Hatch et al. (2020) used molecular data in revising the relevant taxa. The taxonomic groups investigated included Polynoidae, Hesionidae, Siboglinidae, Ampharetidae, Chrysopetalidae, Capitellidae, Orbiniidae and Scalibregmatidae. Representative studies include revisions of Polynoidae by Lindgren et al. (2019), Hatch et al. (2020) and Han et al. (2023); studies of Hesionidae by Rouse et al. (2018a), Shimabukuro et al. (2019) and Kroesche and Rouse (2025); descriptions of Siboglinidae by McCowin and Rouse (2018a) and Xu et al. (2022); phylogenetic analyses of Ampharetidae by Stiller et al. (2013) and Zhou et al. (2019); records of Chrysopetalidae by Watson et al. (2016); and revisions of Orbiniidae and Scalibregmatidae by Blake (2020, 2025). In addition, Seid et al. (2025) published a faunal inventory of a methane seep off Costa Rica and Georgieva et al. (2023a) reported a whale-fall community in Byron Bay, Australia. Hiley et al. (2025) described seven new species of Lepidonotopodini. Han et al. (2023) and Sun et al. (2025) described new species from hydrothermal vents in the Indian Ocean, while Carrerette et al. (2026) described Amphisamytha from the Okinawa Trough. The habitats represented in these references include hydrothermal vents, cold seeps, whale falls, wood falls and experimental substrates. Their geographical coverage further expanded across the Western Pacific, Atlantic, Indian and Southern oceans.

Overall, these references provide the taxonomic and systematic foundation for the 368 taxonomic records in the dataset. Their temporal coverage captures the accumulation of knowledge on polychaetes inhabiting deep-sea chemosynthetic and reducing habitats, from traditional morphology-based taxonomy to the integration of molecular phylogenetics and morphology. Over time, the literature has expanded in both taxonomic and geographical scope, encompassing hydrothermal vents, cold seeps, whale falls, wood falls and other reducing habitats across the major ocean basins.

### Data reuse and usage notes

This dataset can be used to query current accepted names and taxonomic status, compile regional or thematic checklists, summarise global or regional taxonomic composition, conduct broad-scale habitat-association comparisons and serve as a taxonomic backbone for occurrence-level data cleaning and data mobilisation. 'AphiaID', 'scientificNameID' (LSID) and 'referenceIDs' facilitate linkages to WoRMS, OBIS, GBIF and institutional specimen databases. The data may also be used for bibliometrics, knowledge graphs and AI-assisted information extraction, but such applications should still retain a step of verifying against original literature.

When using the data, it should be clearly understood that the main table is checklist-level: each row represents a taxon, not a station, sampling event or individual. Core statistics are recommended to be filtered by 'includeInCoreStatistics', combined with sensitivity analyses using 'recordType', 'taxonStatus' and 'habitatCertainty'. Multi-valued 'habitatStandardized' indicates that the same taxon has been reported from multiple habitats; when counting habitats, the multi-value field should be split and deduplicated by unique taxon identifiers. For spatial analyses, users should return to original literature or occurrence-level data sources to verify coordinates, depth and sampling time.

### Limitations

The dataset relies on published literature and its completeness is affected by search coverage, the degree of digitisation of historical literature and language accessibility. Thus, version 1.0.1 represents the best available information at the time of release, not an absolutely exhaustive global checklist. The main table lacks systematic coordinates, sampling dates, depths, specimen numbers and abundance information and cannot be directly used for fine-scale species distribution modelling, station-level community analysis, sampling-effort correction or temporal studies. Some habitat associations derive from subsequent handbooks or reviews and the definitions and successional stages of different habitats may vary. Taxonomic status will continue to change with new evidence; accepted status should not be treated as a final conclusion. Widespread species, cf. records and suspected additional taxa especially require cautious interpretation in light of the latest taxonomic research.

### Maintenance and versioning

The dataset is maintained with versioned updates. Future updates may include new species and new records, changes in WoRMS taxonomic status, supplementation of overlooked literature, re-assessment of habitat assignments, field improvements and expansion to occurrence-level data. Patch versions are used for format or spelling corrections only; minor versions for the addition of records or partial field adjustments; major versions for substantial changes to data structure, inclusion criteria or core framework. Each update should list additions, deletions and revisions in the CHANGELOG, retaining original names and modification justifications to maintain inter-version traceability.

## Figures and Tables

**Table 1.** Files included in dataset version 1.0.1 and their contents.

**File**	**Description**
taxon_checklist.csv	Main checklist table: taxonomy, WoRMS identifiers, habitats, locations, references and notes
references.csv	Source reference table, corresponding to the referenceIDs field
habitat_vocabulary.csv	Standardised habitat controlled vocabulary and definitions
field_dictionary.csv	Field definitions and filling rules for the main checklist table
taxonomic_notes.csv	Notes on provisional identifications, taxonomic issues, habitat uncertainty and suspected additional taxa
global_checklist_polychaetes_chemosynthetic_habitats.xlsx	Human-readable Excel workbook
README.md	Data structure, processing rules and recommended usage
CITATION.cff	Machine-readable citation metadata
LICENSE.txt	CC BY 4.0 licence
CHANGELOG.md	Version update changelog

## References

[B14527408] Aguado M. Teresa, Rouse Greg W. (2011). Nautiliniellidae (Annelida) from Costa Rican cold seeps and a western Pacific hydrothermal vent, with description of four new species. Systematics and Biodiversity.

[B14526430] Alalykina Inna L., Polyakova Neonila E. (2022). New species of *Ophryotrocha* (Annelida: Dorvilleidae) associated with deep-sea reducing habitats in the Bering Sea, northwest Pacific. Deep Sea Research Part II: Topical Studies in Oceanography.

[B14527972] Andersen Ann C, Hourdez S, Marie B, Jollivet D, Lallier F H, Sibuet M (2004). *Escarpia
southwardae* sp. nov., a new species of vestimentiferan tubeworm (Annelida, Siboglinidae) from west African cold seeps. Canadian Journal of Zoology.

[B14527093] Artüz M. Levent, Sönmez Bariş, Kubanç Cüneyt (2014). First record of *Phyllochaetopterus
socialis* aggregates (Annelida: Polychaeta) from a hydrothermal vent site in the Sea of Marmara, Turkey. Marine Biodiversity Records.

[B14527818] Bastida-Zavala J. Rolando (2008). Serpulids (Annelida: Polychaeta) from the eastern Pacific, including a brief mention of Hawaiian serpulids. Zootaxa.

[B14526212] Bergamo Gilberto, Carrerette Orlemir, Shimabukuro Mauricio, Santos Cinthya S G, Sumida Paulo Y G (2023). Revealing a new eyeless *Nereis* (Nereididae: Annelida) clade from deep-sea organic falls. Zoological Journal of the Linnean Society.

[B14410292] Bienhold Christina, Pop Ristova Petra, Wenzhöfer Frank, Dittmar Thorsten, Boetius Antje (2013). How deep-sea wood falls sustain chemosynthetic life. PLOS ONE.

[B14531140] Blake James A. (1985). Polychaeta from the vicinity of deep-sea geothermal vents in the eastern Pacific. I. Euphrosinidae, Phyllodocidae, Hesionidae, Nereididae, Glyceridae, Dorvilleidae, Orbiniidae, and Maldanidae. Bulletin of the Biological Society of Washington.

[B14531041] Blake James A. (1990). A new genus and species of Polychaeta commensal with a deep-sea thyasirid clam. Proceedings of the Biological Society of Washington.

[B14531032] Blake James A. (1991). A new species of *Hesiocaeca* (Polychaeta: Hesionidae) from hydrothermal vents at the Mariana Back-Arc Basin with notes on other polychaetes. Proceedings of the Biological Society of Washington.

[B14530977] Blake James A. (1993). New genera and species of deep-sea polychaetes of the family Nautiliniellidae from the Gulf of Mexico and the eastern Pacific. Proceedings of the Biological Society of Washington.

[B14530867] Blake James (2000). A new genus and species of polychaete worm (Family Orbiniidae) from methane seeps in the Gulf of Mexico, with a review of the systematics and phylogenetic interrelationships of the genera of Orbiniidae. Cahiers de Biologie Marine.

[B14527496] Blake James A. (2009). Redescription of *Capitella
capitata*（Fabricius) from West Greenland and designation of a neotype (Polychaeta, Capitellidae). Zoosymposia.

[B14526466] Blake James A. (2020). New species and records of deep-water Orbiniidae (Annelida, Polychaeta) from the eastern Pacific continental slope, abyssal Pacific Ocean, and the South China Sea. Zootaxa.

[B14526486] Blake James A., Ramey-Balcı P. A. (2020). A new genus and species of spionid polychaete (Annelida, Spionidae) from a deep-water cold seep site in the eastern Mediterranean Sea off Turkey. Zoosymposia.

[B14526062] Blake James A. (2025). New species and records of Scalibregmatidae (Annelida) from the Atlantic Ocean, Indian Ocean, Pacific Ocean, Southern Ocean, and adjacent seas. Megataxa.

[B14527590] Böggemann Markus (2009). Polychaetes (Annelida) of the abyssal SE Atlantic. Organisms Diversity & Evolution.

[B14527285] Borda Elizabeth, Kudenov Jerry D., Chevaldonné Pierre, Blake James A., Desbruyères Daniel, Fabri Marie-Claire, Hourdez Stéphane, Pleijel Fredrik, Shank Timothy M., Wilson Nerida G., Schulze Anja, Rouse Greg W. (2013). Cryptic species of *Archinome* (Annelida: Amphinomida) from vents and seeps. Proceedings of the Royal Society B: Biological Sciences.

[B14551138] Bright Monika, Lallier François, Gibson R. N., Atkinson R. J. A., Gordon J. D. M. (2010). Oceanography and Marine Biology: An Annual Review.

[B14526111] Carrerette Orlemir, Bergamo Gilberto, Shimabukuro Maurício, Jimi Naoto, Fujiwara Yoshihiro, Sumida Paulo Y. G. (2026). Hitchhiking in the deep: a new *Amphisamytha* species (Annelida: Ampharetidae) riding on widespread squat lobster at hydrothermal vents in the Okinawa Trough (NW Pacific). Deep Sea Research Part I: Oceanographic Research Papers.

[B14526277] Chen Chong, Jamieson John W., Tunnicliffe Verena (2024). Hydrothermal vent fauna of the Galápagos Rift: updated species list with new records. Marine Biodiversity.

[B14410311] Corliss John B., Dymond Jack, Gordon Louis I., Edmond John M., von Herzen Richard P., Ballard Robert D., Green Kenneth, Williams David, Bainbridge Arnold, Crane Kathy, van Andel Tjeerd H. (1979). Submarine thermal springs on the Galápagos Rift. Science.

[B14527995] Dahlgren Thomas G., Glover Adrian G., Baco Amy, Smith Craig R. (2004). Fauna of whale falls: systematics and ecology of a new polychaete (Annelida: Chrysopetalidae) from the deep Pacific Ocean. Deep Sea Research Part I: Oceanographic Research Papers.

[B14531178] Davis P. H., Spies R. B. (1980). Infaunal benthos of a natural petroleum seep: study of community structure. Marine Biology.

[B14531122] Desbruyères Daniel, Laubier Lucien (1986). Les Alvinellidae, une famille nouvelle d'annélides polychètes inféodées aux sources hydrothermales sous-marines: systématique, biologie et écologie. Canadian Journal of Zoology.

[B14531113] Desbruyères Daniel, Laubier L. (1988). Exploitation d’une source de matière organique concentrée dans l’océan profond: intervention d’une annélide polychète nouvelle. Compte rendu hebdomaidaire des séances de l’Académie des sciences.

[B14531068] Desbruyères Daniel, Laubier L (1989). *Paralvinella
hessleri*, new species of Alvinellidae (Polychaeta) from the Mariana Back-Arc Basin hydrothermal vents. Proceedings of the Biological Society of Washington.

[B14531486] Desbruyères Daniel, Laubier Lucien (1991). Systematics, phylogeny, ecology and distribution of the Alvinellidae (Polychaeta) from deep-Sea hydrothermal vents. Systematics, Biology and Morphology of World Polychaeta.

[B14530932] Desbruyères D., Toulmond A. (1998). A new species of hesionid worm, *Hesiocaeca
methanicola* sp. nov. (Polychaeta: Hesionidae), living in ice-like methane hydrates in the deep Gulf of Mexico.. Cahiers de Biologie Marine.

[B14410478] Desbruyères Daniel, Segonzac Michel, Bright Monika (2006). Handbook of deep-sea hydrothermal vent fauna.

[B14527986] Dreyer J., Miura T., Van Dover CL (2004). *Vesicomyicola
trifurcatus*, a new genus and species of commensal polychaete (Annelida: Polychaeta: Nautiliniellidae) found in deep-sea clams from the Blake Ridge cold seep. Proceedings of the Biological Society of Washington.

[B14527206] Eichinger Irmgard, Hourdez Stéphane, Bright Monika (2013). Morphology, microanatomy and sequence data of *Sclerolinum
contortum* (Siboglindae, Annelida) of the Gulf of Mexico. Organisms Diversity & Evolution.

[B14526740] Eilertsen Mari H., Kongsrud Jon A., Alvestad Tom, Stiller Josefin, Rouse Greg W., Rapp Hans T. (2017). Do ampharetids take sedimented steps between vents and seeps? Phylogeny and habitat-use of Ampharetidae (Annelida, Terebelliformia) in chemosynthesis-based ecosystems. BMC Evolutionary Biology.

[B14526626] Eilertsen Mari H., Georgieva Magdalena N., Kongsrud Jon A., Linse Katrin, Wiklund Helena, Glover Adrian G., Rapp Hans T. (2018). Genetic connectivity from the Arctic to the Antarctic: *Sclerolinum
contortum* and *Nicomache
lokii* (Annelida) are both widespread in reducing environments. Scientific Reports.

[B14531169] Fauchald K., Hancock D. R. (1981). Deep-water polychaetes from a transect off central Oregon. Allan Hancock Monographs in Marine Biology.

[B14527645] Fiege Dieter, Bock Gordon (2009). A new species of *Archinome* (Polychaeta: Archinomidae) from hydrothermal vents on the Pacific–Antarctic Ridge 37°S. Journal of the Marine Biological Association of the United Kingdom.

[B14527916] Fujikura Katsunori, Fujiwara Yoshihiro, Kawato Masaru (2006). A new species of *Osedax* (Annelida: Siboglinidae) associated with whale carcasses off Kyushu, Japan. Zoological Science.

[B14530847] Gardiner S. L., McMullin E., Fisher C. R. (2001). *Seepiophila
jonesi*, a new genus and species of vestimentiferan tube worm (Annelida : Pogonophora) from hydrocarbon seep communities in the Gulf of Mexico. Proceedings of the Biological Society of Washington.

[B14528236] Gardiner S. L., Hourdez Stéphane (2003). On the occurrence of the vestimentiferan tube worm *Lamellibrachia
luymesi* van der Land and Nørrevang, 1975 (Annelida: Pogonophora) in hydrocarbon seep communities in the Gulf of Mexico. Proceedings of the Biological Society of Washington.

[B14526859] Gaudron S. M., Hourdez S., Olu K. (2017). Aspects on gametogenesis, fertilization and embryogenesis of two deep-sea polychaetes from eastern Atlantic cold seeps. Deep Sea Research Part I: Oceanographic Research Papers.

[B14410619] GBIF.org GBIF Home Page. https://www.gbif.org.

[B14527018] Georgieva Magdalena N., Wiklund Helena, Bell James B., Eilertsen Mari H., Mills Rachel A., Little Crispin T. S., Glover Adrian G. (2015). A chemosynthetic weed: the tubeworm *Sclerolinum
contortum* is a bipolar, cosmopolitan species. BMC Evolutionary Biology.

[B14526371] Georgieva Magdalena N., Wiklund Helena, Ramos Dino A., Neal Lenka, Glasby Christopher J., Gunton Laetitia M. (2023). The annelid community of a natural deep-sea whale fall off eastern Australia. Records of the Australian Museum.

[B14526317] Georgieva Magdalena N., Rimskaya-Korsakova Nadezhda N., Krolenko Varvara I., Van Dover Cindy Lee, Amon Diva J., Copley Jonathan T., Plouviez Sophie, Ball Bernard, Wiklund Helena, Glover Adrian G. (2023). A tale of two tubeworms: taxonomy of vestimentiferans (Annelida: Siboglinidae) from the Mid-Cayman Spreading Centre. Invertebrate Systematics.

[B14527963] Glover Adrian G., Goetze Erica, Dahlgren Thomas G., Smith Craig R. (2005). Morphology, reproductive biology and genetic structure of the whale‐fall and hydrothermal vent specialist, *Bathykurila
guaymasensis* Pettibone, 1989 (Annelida: Polynoidae). Marine Ecology.

[B14526840] Goffredi Shana K., Johnson Shannon, Tunnicliffe Verena, Caress David, Clague David, Escobar Elva, Lundsten Lonny, Paduan Jennifer B., Rouse Greg, Salcedo Diana L., Soto Luis A., Spelz-Madero Ronald, Zierenberg Robert, Vrijenhoek Robert (2017). Hydrothermal vent fields discovered in the southern Gulf of California clarify role of habitat in augmenting regional diversity. Proceedings of the Royal Society B: Biological Sciences.

[B14526439] Han Yuru, Zhang Dongsheng, Wang Chunsheng, Zhou Yadong (2021). Out of the Pacific: a new alvinellid worm (Annelida: Terebellida) from the northern Indian Ocean hydrothermal vents. Frontiers in Marine Science.

[B14526268] Han Yuru, Zhou Yadong, Chen Chong, Wang Yueyun (2023). Diversity and biogeography of scale worms in the subfamily Lepidonotopodinae (Annelida: Polynoidae) from Indian Ocean hydrothermal vents with descriptions of four new species. Zoological Journal of the Linnean Society.

[B14531196] Hartmann-Schröder G. (1977). Die Polychaeten der Kubanisch-Rumänischen Biospeologischen Expedition nach Kuba 1973. Résultats des expéditions biospéologiques cubano-roumaines à Cuba.

[B14531280] Hartman Olga (1957). Orbiniidae, Apistobranchidae, Paraonidae and Longosomidae. Allan Hancock Pacific Expeditions.

[B14531407] Hatch Avery S., Liew Haebin, Hourdez Stéphane, Rouse Greg W. (2020). Hungry scale worms: phylogenetics of *Peinaleopolynoe* (Polynoidae, Annelida), with four new species. ZooKeys.

[B14410366] Hilário Ana, Capa María, Dahlgren Thomas G., Halanych Kenneth M., Little Crispin T. S., Thornhill Daniel J., Verna Caroline, Glover Adrian G. (2011). New perspectives on the ecology and evolution of siboglinid tubeworms. PLOS ONE.

[B14531445] Hilbig Brigitte, Blake James A., Hilbig Brigitte, Scott Paul H. (1997). Taxonomic Atlas of the Benthic Fauna of the Santa Maria Basin and Western Santa Barbara Channel, Volume 4, The Annelida, Part 1. Oligochaeta and Polychaeta: Phyllodocida (Phyllodocidae to Paralacydoniidae).

[B14526140] Hiley Avery S., Green Kiirah R., Rouse Greg W. (2025). Seven new species of scaleworms (Lepidonotopodini, Polynoidae, Polychaeta, Annelida) from deep-sea chemosynthetic-based ecosystems. Marine Biodiversity.

[B14528280] Hourdez Stéphane, Daniel Desbruyeres (2003). A new species of scale-worm (Polychaeta : Polynoidae), *Levensteiniella
iris* sp. nov., from the Rainbow and Lucky Strike vent fields (Mid-Atlantic Ridge). Cahiers de Biologie Marine.

[B14526233] Hui F., Lin Y. T., Perez M., Qiu J. W., Sun Y. (2024). A new species of *Natsushima* (Annelida: Chrysopetalidae) living in the mantle cavity of a deep-sea solemyid clam. Zoological Studies.

[B14527127] Imajima Minoru, Reuscher Michael G., Fiege Dieter (2013). Ampharetidae (Annelida: Polychaeta) from Japan. Part II: genera with elevated and modified notopodia. Zootaxa.

[B14530995] Ivanov A. V., Selivanova R. V. (1992). *Sclerolinum
javanicum* sp. n., a new pogonophoran living on rotten wood. A contribution to the classification of Pogonophora. Biologiya Morya (Vladivostok).

[B14526617] Jimi Naoto, Chen Chong, Hiroshi Kajihara (2019). New record of the hydrothermal vent-endemic polychaete *Archinome
jasoni* Borda et al., 2013 (Annelida, Amphinomidae) from the northwest Pacific. Check List.

[B14526421] Jimi Naoto, Chen Chong, Fujiwara Yoshihiro (2022). Two new species of *Branchinotogluma* (Polynoidae: Annelida) from chemosynthesis-based ecosystems in Japan. Zootaxa.

[B14526305] Kamei Haruka, Okamoto Kai, Ohta Mizuki, Itoh Hajime, Watanabe Hiromi Kayama, Komatsu Hironori, Kojima Shigeaki (2023). Discovery and genetic characterization of a Vestimentiferan, *Lamellibrachia
satsuma*, from the Submarine Volcano Omuro Dashi in the Izu-Ogasawara Arc. Zoological Science.

[B14526990] Karaseva N. P., Rimskaya-Korsakova N. N., Galkin S. V., Malakhov V. V. (2017). Taxonomy, geographical and bathymetric distribution of vestimentiferan tubeworms (Annelida, Siboglinidae). Biology Bulletin.

[B14526411] Kim Sang Lyeol, Choi Hyeongwoo, Eyun Seong-il, Kim Dongsung, Yu Ok Hwan (2022). A new *Branchipolynoe* (Aphroditiformia: Polynoidae) scale worm from the Onnuri deep-sea hydrothermal vent field, Northern Central Indian Ridge. Zoological studies.

[B14531004] Kiseleva M. I. (1992). New genus and species of a polychaete of the Chrysopetalidae from the Black Sea. Zoologicheskii Zhurnal.

[B14526661] Kobayashi Genki, Araya Juan Francisco (2018). Southernmost records of *Escarpia
spicata* and *Lamellibrachia
barhami* (Annelida: Siboglinidae) confirmed with DNA obtained from dried tubes collected from undiscovered reducing environments in northern Chile. PLOS ONE.

[B14528289] Kojima Shigeaki, Ohta Shigeo, Yamamoto Tetsuya, Miura Toshihiko, Fujiwara Yoshihiro, Fujikura Koji, Hashimoto Jun (2002). Molecular taxonomy of vestimentiferans of the western Pacific and their phylogenetic relationship to species of the eastern Pacific. Marine Biology.

[B14527934] Kojima Shigeaki, Watanabe Hiromi, Tsuchida Shinji, Fujikura Katsunori, Rowden Ashley A., Takai Ken, Miura Tomoyuki (2006). Phylogenetic relationships of a tube worm from three hydrothermal vent fields in the south Pacific. Journal of the Marine Biological Association of the United Kingdom.

[B14527311] Kongsrud Jon Anders, Rapp Hans Tore (2011). Nicomache (Loxochona) lokii sp. nov. (Annelida: Polychaeta: Maldanidae) from the Loki’s Castle vent field: an important structure builder in an Arctic vent system. Polar Biology.

[B14526720] Kongsrud Jon Anders, Eilertsen Mari H., Alvestad Tom, Kongshavn Katrine, Rapp Hans Tore (2017). New species of Ampharetidae (Annelida: Polychaeta) from the Arctic Loki Castle vent field. Deep Sea Research Part II: Topical Studies in Oceanography.

[B14526092] Kroesche Rachel, Rouse Greg W. (2025). Four new species of Hesionidae (Annelida, Polychaeta, Phyllodocida) from eastern Pacific chemosynthetic habitats and reinstatement of *Vrijenhoekia*. Diversity.

[B14530968] Kupriyanova Elena K. (1993). Deep-water Serpulidae (Annelida, Polychaeta) from the Kurile-Kamchatka trench: 1. Genus *Hyalopomatus*. Zoologichesky Zhurnal.

[B14527449] Kupriyanova Elena K., Nishi Eiljiroh, Kawato Masaru, Fujiwara Yoshihiro (2010). New records of Serpulidae (Annelida, Polychaeta) from hydrothermal vents of North Fiji, Pacific Ocean. Zootaxa.

[B14531104] Kuznetsov A. P., Levenstein R. Y. (1988). *Pavelius
uschakovi* gen. et sp. n. (Polychaeta, Ampharetidae) from Paramushir gas hydrate spring in the Okhotsk Sea.. Zoologicheskii Zhurnal.

[B14531205] Land Jacob van der, Nørrevang Arne (1975). The systematic position of *Lamellibrachia* (Annelida, Vestimentifera). Zeitschrift für Zoologische Systematik und Evolutionsforschung.

[B14410388] Levin Lisa A., Barry James P., Felbeck Horst, Smith Craig R., Young Craig M. (2007). Advances in vent, seep, whale‐ and wood‐fall biology. Marine Ecology.

[B14526607] Lindgren Johanna, Hatch Avery S., Hourdez Stephané, Seid Charlotte A., Rouse Greg W. (2019). Phylogeny and biogeography of *Branchipolynoe* (Polynoidae, Phyllodocida, Aciculata, Annelida), with descriptions of five new species from methane seeps and hydrothermal vents. Diversity.

[B14526382] Magalhães Wagner F., Hilliard Justin (2022). Two new deep-sea species of *Capitella* (Annelida: Capitellidae) from sunken wood in the northeast Pacific. Zootaxa.

[B14531131] Mañé-Garzón F., Montero R. (1985). Sobre una nueva forma de verme tubicola *Lamellibrachia
victori* n. sp. (Vestimentifera), proposition de un nuevo phylum: Mesoneurophora. Revista de Biologia del Uruguay.

[B14526711] McCowin Marina F., Rouse Greg W. (2018). Phylogeny of hydrothermal vent Iphionidae, with the description of a new species (Aphroditiformia, Annelida). ZooKeys.

[B14526652] McCowin Marina F., Rouse Greg W. (2018). A new *Lamellibrachia* species and confirmed range extension for *Lamellibrachia
barhami* (Siboglinidae, Annelida) from Costa Rica methane seeps. Zootaxa.

[B14526548] McCowin Marina F., Rowden Ashley A., Rouse Greg W. (2019). A new record of *Lamellibrachia
columna* (Siboglinidae, Annelida) from cold seeps off New Zealand, and an assessment of its presence in the western Pacific Ocean. Marine Biodiversity Records.

[B14526332] McCowin Marina F., Collins Patrick C., Rouse Greg W. (2023). Updated phylogeny of Vestimentifera (Siboglinidae, Polychaeta, Annelida) based on mitochondrial genomes, with a new species. Molecular Phylogenetics and Evolution.

[B14528271] McMullin E. R., Hourdez S., Schaeffer S. W., Fisher C. R. (2003). Phylogeny and biogeography of deep sea vestimentiferan tubeworms and their bacterial symbionts. Symbiosis.

[B14526182] Meca Miguel A., Kongsrud Jon Anders, Kongshavn Katrine, Alvestad Tom, Meißner Karin, Budaeva Nataliya (2024). Diversity of *Orbiniella* (Orbiniidae, Annelida) in the north Atlantic and the Arctic. ZooKeys.

[B14531014] Miura T., Hashimoto J. (1991). *Nicomache
ohtai*, new species (Polychaeta: Maldanidae) collected from the Hatsushima cold-seep in Sagami Bay. Proceedings of the Biological Society of Washington.

[B14530959] Miura Tomoyuki, Hashimoto Jun (1996). Nautiliniellid polychaetes living in the mantle cavity of bivalve mollusks from cold seeps and hydrothermal vents around Japan. Publications of the Seto Marine Biological Laboratory.

[B14530950] Miura T. (1997). Two new species of the genus *Ophryotrocha* (Polychaeta, Iphitimiidae) from Kagoshima Bay. Bulletin of Marine Science.

[B14527925] Miura Tomoyuki, Kojima Shigeaki (2006). Two new species of vestimentiferan tubeworm (Polychaeta: Siboglinidae a.k.a. Pogonophora) from the Brothers Caldera, Kermadec Arc, South Pacific Ocean. Species Diversity.

[B14530910] Nishi E., Miura T., Bhaud Michel (1999). A new species of *Spiochaetopterus* (Chaetopteridae : Polychaeta) from a cold-seep site off Hatsushima in Sagami Bay, Central Japan. Proceedings of the Biological Society of Washington.

[B14527898] Nishi Eijiroh, Rouse Greg W. (2007). A new species of *Phyllochaetopterus* (Chaetopteridae: Annelida) from near hydrothermal vents in the Lau Basin, western Pacific Ocean. Zootaxa.

[B14527699] Nishi Eijiroh (2008). A new species of *Spiochaetopterus* (Chaetopteridae: Polychaeta) from the Iheya Seamount off Okinawa in East China Sea, Japan. Proceedings of the Biological Society of Washington.

[B14410627] OBIS Ocean Biodiversity Information System. https://obis.org.

[B14531187] Oug Eivind (1978). New and lesser known Dorvilleidae (Annelida, Polychaeta) from Scandinavian and northeast American waters. Sarsia.

[B14410398] Paull C. K., Hecker B., Commeau R., Freeman-Lynde R. P., Neumann C., Corso W. P., Golubic S., Hook J. E., Sikes E., Curray J. (1984). Biological communities at the Florida Escarpment resemble hydrothermal vent taxa. Science.

[B14527626] Paxton Hannelore, Morineaux Marie (2009). Three species of Dorvilleidae (Annelida: Polychaeta) associated with Atlantic deep-sea reducing habitats, with the description of *Ophryotrocha
fabriae*, new species. Proceedings of the Biological Society of Washington.

[B14526402] Pearson Kailaa. M., Rouse Greg W. (2022). Vampire worms; a revision of *Galapagomystides* (Phyllodocidae, Annelida), with the description of three new species. Zootaxa.

[B14531262] Pettibone Marian H. (1957). North American genera of the family Orbiniidae (Annelida: Polychaeta), with descriptions of new species. Journal of the Washington Academy of Sciences.

[B14531149] Pettibone Marian H. (1985). New genera and species of deep-sea Macellicephalinae and Harmothoinae (Polychaeta: Polynoidae) from the hydrothermal rift areas off the Galapagos and western Mexico at 21°N and from the Santa Catalina Channel. Proceedings of the Biological Society of Washington.

[B14531086] Pettibone Marian H. (1989). Polynoidae and Sigalionidae (Polychaeta) from the Guaymas Basin, with descriptions of two new species, and additional records from hydrothermal vents of the Galapagos Rift, 21°N, and seep sites in the Gulf of Mexico (Florida and Louisiana). Proceedings of the Biological Society of Washington.

[B14531077] Pettibone Marian H. (1989). New species of scale-worms (Polychaeta:Polynoidae) from the hydrothermal rift-area of the Mariana Back-Arc Basin in the Western Central Pacific. Proceedings of the Biological Society of Washington.

[B14531059] Pettibone Marian H. (1990). New species and new records of scaled polychaetes (Polychaeta: Polynoidae) from the Axial Seamount Caldera of the Juan de Fuca Ridge in the northeast Pacific and the east Pacific Ocean off northern California. Proceedings of the Biological Society of Washington.

[B14530986] Pettibone Marian H. (1993). Polynoid polychaetes associated with a whale skeleton in the bathyal Santa Catalina Basin. Proceedings of the Biological Society of Washington.

[B14527887] Pleijel Fredrik, Rouse Greg W., Ruta Christine, Wiklund Helena, Nygren Arne (2008). *Vrijenhoekia
balaenophila*, a new hesionid polychaete from a whale fall off California. Zoological Journal of the Linnean Society.

[B14527366] Pleijel Fredrik, Rouse Greg W., Sundkvist Tobias, Nygren Arne (2012). A partial revision of *Gyptis* (Gyptini, Ophiodrominae, Hesionidae, Aciculata, Annelida), with descriptions of a new tribe, a new genus and five new species. Zoological Journal of the Linnean Society.

[B14527039] Plouviez Sophie, Jacobson Alixandra, Wu Mengyou, Van Dover Cindy L. (2015). Characterization of vent fauna at the Mid-Cayman Spreading Center. Deep Sea Research Part I: Oceanographic Research Papers.

[B14526730] Queirós José Pedro, Ravara Ascensão, Eilertsen Mari H., Kongsrud Jon A., Hilário Ana (2017). *Paramytha
ossicola* sp. nov. (Polychaeta, Ampharetidae) from mammal bones: reproductive biology and population structure. Deep Sea Research Part II: Topical Studies in Oceanography.

[B14527617] Quiroga Eduardo, Sellanes Javier (2009). Two new polychaete species living in the mantle cavity of *Calyptogena
gallardoi* (Bivalvia: Vesicomyidae) at a methane seep site off central Chile (~36°S). Scientia Marina.

[B14527458] Quiroga Eduardo, Levin Lisa A (2010). *Eunice
pennata* (Polychaeta: Eunicidae) from active and passive cold seep sites in central and southern Chile (36°–46°S). Anales del Instituto de la Patagonia.

[B14527907] Ravara Ascensao, Cunha Marina R., Rodrigues Clara F. (2007). The occurrence of *Natsushima
bifurcata* (Polychaeta: Nautiliniellidae) in *Acharax* hosts from mud volcanoes in the Gulf of Cadiz (south Iberian and north Moroccan margins). Scientia Marina.

[B14527084] Ravara Ascensão, Marçal Ana R., Wiklund Helena, Hilário Ana (2015). First account on the diversity of *Ophryotrocha* (Annelida, Dorvilleidae) from a mammal-fall in the deep-Atlantic Ocean with the description of three new species. Systematics and Biodiversity.

[B14526830] Ravara Ascensão, Ramos Diana, Teixeira Marcos A. L., Costa Filipe O., Cunha Marina R. (2017). Taxonomy, distribution and ecology of the order Phyllodocida (Annelida, Polychaeta) in deep-sea habitats around the Iberian margin. Deep Sea Research Part II: Topical Studies in Oceanography.

[B14526588] Ravara Ascensão, Aguado M. Teresa, Rodrigues Clara F., Génio Luciana, Cunha Marina R. (2019). Description of a new genus and species of Chrysopetalidae (Annelida: Polychaeta) from the NE Atlantic, with some further records of related species. European Journal of Taxonomy.

[B14526457] Ravara Ascensão, Wiklund Helena, Cunha Marina R. (2021). Four new species and further records of Dorvilleidae (Annelida, Polychaeta) from deep-sea organic substrata, NE Atlantic. European Journal of Taxonomy.

[B14527505] Reuscher Michael, Fiege Dieter, Wehe Thomas (2009). Four new species of Ampharetidae (Annelida: Polychaeta) from Pacific hot vents and cold seeps, with a key and synoptic table of characters for all genera. Zootaxa.

[B14527322] Reuscher Michael, Fiege Dieter, Wehe Thomas (2011). Terebellomorph polychaetes from hydrothermal vents and cold seeps with the description of two new species of Terebellidae (Annelida: Polychaeta) representing the first records of the family from deep-sea vents. Journal of the Marine Biological Association of the United Kingdom.

[B14526981] Reuscher Michael, Fiege Dieter (2016). Ampharetidae (Annelida: Polychaeta) from cold seeps off Pakistan and hydrothermal vents off Taiwan, with the description of three new species. Zootaxa.

[B14410422] Rouse Greg W., Goffredi S. K., Vrijenhoek R. C. (2004). *Osedax*: bone-eating marine worms with dwarf males. Science.

[B14531436] Rouse Greg W., Carvajal Jose Ignacio, Pleijel Fredrik (2018). Phylogeny of Hesionidae (Aciculata, Annelida), with four new species from deep-sea eastern Pacific methane seeps, and resolution of the affinity of *Hesiolyra*. Invertebrate Systematics.

[B14526671] Rouse Greg W., Goffredi Shana K., Johnson Shannon B., Vrijenhoek Robert C. (2018). An inordinate fondness for *Osedax* (Siboglinidae: Annelida): fourteen new species of bone worms from California. Zootaxa.

[B14526448] Rouse Greg W., Kupriyanova Elena K. (2021). *Laminatubus* (Serpulidae, Annelida) from eastern Pacific hydrothermal vents and methane seeps, with description of two new species. Zootaxa.

[B14527102] Rubin-Blum Maxim, Tsadok Rami, Shemesh Eli, Goodman-Tchernov Beverly N., Austin James A., Coleman Dwight F., Ben-Avraham Zvi, Gruber David F., Tchernov Dan (2014). Distribution of the *Lamellibrachia* spp. (Siboglinidae, Annelida) and their trophosome endosymbiont phylotypes in the Mediterranean Sea. Marine Biology.

[B14526557] Saeedi Hanieh, Bernardino Angelo F., Shimabukuro Maurício, Falchetto Giulia, Sumida Paulo Y. G. (2019). Macrofaunal community structure and biodiversity patterns based on a wood-fall experiment in the deep south-west Atlantic. Deep Sea Research Part I: Oceanographic Research Papers.

[B14527946] Salazar-Vallejo S. I., Orensanz J. M. (2006). *Pleijelius
longae* n. gen., n. sp., a remarkable deep water polychaete from the northwestern Atlantic (Polychaeta: Hesionidae). Scientia Marina.

[B14526477] Salazar-Vallejo S. I. (2020). Four new deep-water flabelligerid species from Pacific Costa Rica (Annelida, Sedentaria, Flabelligeridae). Zootaxa.

[B14527116] Salvo Flora, Wiklund Helena, Dufour Suzanne C., Hamoutene Dounia, Pohle Gerhard, Worsaae Katrine (2014). A new annelid species from whalebones in Greenland and aquaculture sites in Newfoundland: *Ophryotrocha
cyclops*, sp. nov. (Eunicida: Dorvilleidae). Zootaxa.

[B14526149] Seid Charlotte A., Hiley Avery S., McCowin Marina F., Carvajal José I., Cha Harim, Ahyong Shane T., Ashford Oliver S., Breedy Odalisca, Eernisse Douglas J., Goffredi Shana K., Hendrickx Michel E., Kocot Kevin M., Mah Christopher L., Miller Allison K., Mongiardino Koch Nicolás, Mooi Rich, O'Hara Timothy D., Pleijel Fredrik, Stiller Josefin, Tilic Ekin, Valentich-Scott Paul, Warén Anders, Wicksten Mary K., Wilson Nerida G., Cordes Erik E., Levin Lisa A., Cortés Jorge, Rouse Greg W. (2025). A faunal inventory of methane seeps on the Pacific margin of Costa Rica. ZooKeys.

[B14526751] Shimabukuro Maurício, Santos Cinthya S. G., Alfaro-Lucas Joan M., Fujiwara Yoshihiro, Sumida Paulo Y. G. (2017). A new eyeless species of *Neanthes* (Annelida: Nereididae) associated with a whale-fall community from the deep southwest Atlantic Ocean. Deep Sea Research Part II: Topical Studies in Oceanography.

[B14526577] Shimabukuro Mauricio, Carrerette Orlemir, Alfaro-Lucas Joan Manel, Rizzo Alexandra Elaine, Halanych Kenneth M., Sumida Paulo Yukio Gomes (2019). Diversity, distribution and phylogeny of Hesionidae (Annelida) colonizing whale falls: new species of *Sirsoe* and connections between ocean basins. Frontiers in Marine Science.

[B14526495] Shimabukuro Maurício, Alfaro-Lucas Joan M., Bernardino Angelo F., Ramos Raissa B., de Mahiques Michel M., Sumida Paulo Yukio Gomes (2020). Chemosynthetic ecosystems on the Brazilian deep-sea margin. Brazilian Marine Biodiversity.

[B14528023] Sigvaldadóttir E., Desbruyères D. (2003). Two new species of Spionidae (Annelida: Polychaeta) from Mid-Atlantic Ridge hydrothermal vents. Cahiers de Biologie Marine.

[B14526903] Silva Camila F., Shimabukuro Maurício, Alfaro-Lucas Joan M., Fujiwara Yoshihiro, Sumida Paulo Y. G., Amaral Antonia C. Z. (2016). A new *Capitella* polychaete worm (Annelida: Capitellidae) living inside whale bones in the abyssal south Atlantic. Deep Sea Research Part I: Oceanographic Research Papers.

[B14530876] Smirnov Roman V. (2000). Two new species of Pogonophora from the Arctic mud volcano off northwestern Norway. Sarsia.

[B14410431] Smith C. R., Kukert H., Wheatcroft R. A., Jumars P. A., Deming J. W. (1989). Vent fauna on whale remains. Nature.

[B14531240] Southward Eve C., Weber Max (1961). Siboga-Expeditie. Uitkomsten op Zoologisch, Botanisch, Oceanographisch en Geologisch gebied verzameld in Nederlandsch Oost-Indië 1899-1900.

[B14527399] Southward Eve C., Andersen Ann C., Hourdez Stéphane (2011). *Lamellibrachia
anaximandri* n. sp., a new vestimentiferan tubeworm (Annelida) from the Mediterranean, with notes on frenulate tubeworms from the same habitat. Zoosystema.

[B14531214] Southward E. C. (1972). On some Pogonophora from the Caribbean and the Gulf of Mexico. Bulletin of Marine Science.

[B14531023] Southward E. C. (1991). Three new species of Pogonophora, including two vestimentiferans, from hydrothermal sites in the Lau Back-arc Basin (Southwest Pacific Ocean). Journal of Natural History.

[B14527145] Stiller Josefin, Rousset Vincent, Pleijel Fredrik, Chevaldonné Pierre, Vrijenhoek Robert C., Rouse Greg W. (2013). Phylogeny, biogeography and systematics of hydrothermal vent and methane seep *Amphisamytha* (Ampharetidae, Annelida), with descriptions of three new species. Systematics and Biodiversity.

[B14526286] Sui Jixing, Dong Dong, Wu Xuwen, Li Xinzheng (2023). A new species of the genus *Lindaspio* Blake & Maciolek, 1992 (Annelida, Spionidae) from a cold seep near Hainan Island, China. ZooKeys.

[B14527048] Summers Mindi, Pleijel Fredrik, Rouse Greg W. (2015). Whale falls, multiple colonisations of the deep, and the phylogeny of Hesionidae (Annelida). Invertebrate Systematics.

[B14526638] Sun Yanan, Liang Qianyong, Sun Jin, Yang Yi, Tao Jun, Liang Jinqiang, Feng Dong, Qiu Jian-Wen, Qian Pei-Yuan (2018). The mitochondrial genome of the deep-sea tubeworm *Paraescarpia
echinospica* (Siboglinidae, Annelida) and its phylogenetic implications. Mitochondrial DNA Part B.

[B14526071] Sun Yanan, Bao Qinghua, Chen Chong, Wu Xuwen, Ju Yuxue, Liao Shili, Zhou Yadong (2025). New species of capitellid polychaetes (Annelida: Sedentaria) add to the unique biodiversity of Indian Ocean hot vents. Zoological Journal of the Linnean Society.

[B14527272] Taboada Sergi, Wiklund Helena, Glover Adrian G., Dahlgren Thomas G., Cristobo Javier, Avila Conxita (2013). Two new Antarctic *Ophryotrocha* (Annelida: Dorvilleidae) described from shallow-water whale bones. Polar Biology.

[B14527075] Taboada Sergi, Bas Maria, Avila Conxita (2014). A new *Parougia* species (Annelida, Dorvilleidae) associated with eutrophic marine habitats in Antarctica. Polar Biology.

[B14526923] Taboada Sergi, Bas Maria, Leiva Carlos, Garriga Mariona, Sardá Rafael, Avila Conxita (2015). Life after death: shallow‐water Mediterranean invertebrate communities associated with mammal bones. Marine Ecology.

[B14527340] Thiel Vera, Hügler Michael, Blümel Martina, Baumann Heike I., Gärtner Andrea, Schmaljohann Rolf, Strauss Harald, Garbe-Schönberg Dieter, Petersen Sven, Cowart Dominique A., Fisher Charles R., Imhoff Johannes F. (2012). Widespread occurrence of two carbon fixation pathways in tubeworm endosymbionts: lessons from hydrothermal vent associated tubeworms from the Mediterranean Sea. Frontiers in Microbiology.

[B14526202] Villalobos-Guerrero Tulio F., Huč Sonja, Tilic Ekin, Hiley Avery S., Rouse Greg W. (2024). A remarkable new deep-sea nereidid (Annelida: Nereididae) with gills. PLOS ONE.

[B14526193] Wang Yueyun, Zhou Yadong, Zhang Dongsheng, Wang Chunsheng (2024). Are maldanids from deep-sea reduced habitats closely related? Implications of a new wood-fall species of *Nicomache* from the South China Sea. Frontiers in Marine Science.

[B14526506] Wang Zhi, Xu Ting, Zhang Yanjie, Zhou Yadong, Liu Zhensheng, Chen Chong, Watanabe Hiromi Kayama, Qiu Jian-Wen (2020). Molecular phylogenetic and morphological analyses of the ‘monospecific’ *Hesiolyra* (Annelida: Hesionidae) reveal two new species. Deep Sea Research Part I: Oceanographic Research Papers.

[B14527419] Watanabe Hiromi, Fujikura Katsunori, Kojima Shigeaki, Miyazaki Jun-Ichi, Fujiwara Yoshihiro (2010). Japan: vents and seeps in close proximity. Topics in Geobiology.

[B14530858] Watson Charlotte (2001). New genus and species of Chrysopetalidae (Polychaeta) from hydrothermal vents (South-Western Pacific). The Beagle: Records of the Museums and Art Galleries of the Northern Territory.

[B14527008] Watson Charlotte, Ignacio Carvajal J., Sergeeva Nelly G., Pleijel Fredrik, Rouse Greg W. (2016). Free-living calamyzin chrysopetalids (Annelida) from methane seeps, anoxic basins, and whale falls. Zoological Journal of the Linnean Society.

[B14526258] Watson Charlotte, Gunton Laetitia M., Kupriyanova Elena K. (2024). Three new species of bacterivorous Chrysopetalidae and Microphthalmidae (Annelida) inhabiting a whale fall off eastern Australia. Records of the Australian Museum.

[B14531223] Webb Michael (1965). Notes on the distribution of Pogonophora in Norwegian fjords. Sarsia.

[B14410441] Weiss R. F, Lonsdale P., Lupton J. E., Bainbridge A. E., Craig H. (1977). Hydrothermal plumes in the Galapagos Rift. Nature.

[B14527636] Wiklund Helena, Glover Adrian G., Dahlgren Thomas G. (2009). Three new species of *Ophryotrocha* (Annelida: Dorvilleidae) from a whale-fall in the north-east Atlantic. Zootaxa.

[B14527608] Wiklund Helena, Glover Adrian G., Johannessen Per J., Dahlgren Thomas G. (2009). Cryptic speciation at organic-rich marine habitats: a new bacteriovore annelid from whale-fall and fish farms in the north-east Atlantic. Zoological Journal of the Linnean Society.

[B14527377] Wiklund Helena, Altamira Iris V., Glover Adrian G., Smith Craig R., Baco Amy R., Dahlgren Thomas G. (2012). Systematics and biodiversity of *Ophryotrocha* (Annelida, Dorvilleidae) with descriptions of six new species from deep-sea whale-fall and wood-fall habitats in the north-east Pacific. Systematics and Biodiversity.

[B14410669] Board WoRMS Editorial World Register of Marine Species. https://www.marinespecies.org.

[B14526681] Wu Xuwen, Xu Kuidong (2018). *Levensteiniella
manusensis* sp. nov., a new polychaete species (Annelida: Polynoidae) from deep-sea hydrothermal vents in the Manus Back-Arc Basin, Western Pacific. Zootaxa.

[B14526598] Wu Xuwen, Zhan Zifeng, Xu Kuidong (2019). Two new and two rarely known species of *Branchinotogluma* (Annelida: Polynoidae) from deep-sea hydrothermal vents of the Manus Back-Arc basin, with remarks on the diversity and biogeography of vent polynoids. Deep Sea Research Part I: Oceanographic Research Papers.

[B14526352] Wu Xuwen, Zhen Wenquan, Kou Qi, Xu Kuidong (2023). Reevaluation of the systematic status of *Branchinotogluma* (Annelida, Polynoidae), with the establishment of two new species. Journal of Zoological Systematics and Evolutionary Research.

[B14526391] Xu Ting, Sun Yanan, Wang Zhi, Sen Arunima, Qian Pei-Yuan, Qiu Jian-Wen (2022). The morphology, mitogenome, phylogenetic position, and symbiotic bacteria of a new species of *Sclerolinum* (Annelida: Siboglinidae) in the South China Sea. Frontiers in Marine Science.

[B14526528] Yen Nicole K., Rouse Greg W. (2020). Phylogeny, biogeography and systematics of Pacific vent, methane seep, and whale-fall *Parougia* (Dorvilleidae : Annelida), with eight new species. Invertebrate Systematics.

[B14526885] Zhang Dong-sheng, Zhou Ya-dong, Wang Chun-sheng, Rouse Greg (2017). A new species of *Ophryotrocha* (Annelida, Eunicida, Dorvilleidae) from hydrothermal vents on the Southwest Indian Ridge. ZooKeys.

[B14526361] Zhang Dongsheng, Zhou Yadong, Yen Nicole, Hiley Avery S., Rouse Greg W. (2023). *Ophryotrocha* (Dorvilleidae, Polychaeta, Annelida) from deep-sea hydrothermal vents, with the description of five new species. European Journal of Taxonomy.

[B14526701] Zhang Yanjie, Chen Chong, Qiu Jian-Wen (2018). Sexually dimorphic scale worms (Annelida: Polynoidae) from hydrothermal vents in the Okinawa Trough: two new species and two new sex morphs. Frontiers in Marine Science.

[B14526876] Zhou Yadong, Zhang Dongsheng, Lu Bo, Wang Chunsheng (2017). Description of a new branchiate scale-worm (Polychaeta: Polynoidae) from the hydrothermal vent on Southwest Indian Ocean Ridge. Zootaxa.

[B14526690] Zhou Yadong, Wang Yueyun, Zhang Dongsheng, Wang Chunsheng (2018). *Branchinotogluma
bipapillata* n. sp., a new branchiate scale worm (Annelida: Polynoidae) from two hydrothermal fields on the Southwest Indian Ridge. Zootaxa.

[B14526537] Zhou Yadong, Chen Chong, Sun Yanan, Watanabe Hiromi Kayama, Zhang Ruiyan, Wang Chunsheng (2019). *Amphisamytha* (Annelida: Ampharetidae) from Indian Ocean hydrothermal vents: biogeographic implications. Deep Sea Research Part I: Oceanographic Research Papers.

[B14526341] Zhou Yadong, Han Yuru, Xie Wei, Li Mingting, Wang Zhi, Zhang Dongsheng (2023). Diversity, phylogeny, and bathymetric zonation of *Sirsoe* (Annelida: Hesionidae) from colonization experiments in the South China Sea, with the description of three new species. Ecology and Evolution.

[B14531160] Zottoli Robert (1982). Two new genera of deep sea polychaete worms of the family Ampharetidae and the role of one species in deep sea ecosystems. Proceedings of the Biological Society of Washington.

